# Prevalence of erectile dysfunction and possible risk factors among men of South-Western Nigeria: a population based study

**DOI:** 10.11604/pamj.2016.24.124.8660

**Published:** 2016-06-08

**Authors:** Bolaji Oyetunde Oyelade, Abiodun Christopher Jemilohun, Sunday Adedeji Aderibigbe

**Affiliations:** 1 Ladoke Akintola University of Technology Health Centre, Ogbomoso, Oyo State, Nigeria; 2 Department of Medicine, Ladoke Akintola University of Technology/LAUTECH Teaching Hospital, Ogbomoso, Oyo State, Nigeria; 3 Department of Community Medicine, University of Ilorin/University of Ilorin Teaching Hospital, Ilorin, Kwara, Nigeria

**Keywords:** Erectile dysfunction, Risk factors, Diabetes mellitus, Nigeria

## Abstract

**Introduction:**

Erectile dysfunction (ED) is currently one of the most common sexual dysfunctions worldwide but it is usually underestimated because it is not a life threatening condition. The associated stigma makes men who have it to suffer in silence. This study was conducted to determine the prevalence of erectile dysfunction and the possible associated risk factors among Nigerian men.

**Methods:**

The study was a descriptive cross-sectional population based survey among men aged 30-80 years in Ogbomoso, South-west, Nigeria. A multistage random sampling method was used. The instrument used was the International Index of Erectile Function Questionnaire-5 (IIEF-5). Unadjusted odds ratios of possible risk factors were calculated by univariate analyses. Binary logistic regression analysis was used to eliminate the effect of possible confounders on the risk factors to get the adjusted odds ratios.

**Results:**

The general prevalence of ED in this study was 58.9%. Sixty-seven (47.2%), 16 (11.3%) and 59(41.5%) respondents had mild, moderate and severe ED respectively. Age, hypertension, use of anti-hypertensive drugs, diabetes mellitus and heart disease all had significant unadjusted associations with ED, but their adjusted associations were not statistically significant. Diabetes mellitus maintained a positive statistically significant relationship with ED after adjustment for potential confounders [OR= 8.31(95% CI 1.02 - 67.65), P= 0.048].

**Conclusion:**

The prevalence of ED is high among south-western Nigeria male adults. Physicians, especially primary care ones, need to pay more attention to the sexual history of their patients in order to diagnose and manage ED more frequently.

## Introduction

Erectile dysfunction (ED) is defined as the persistent inability to achieve and maintain an erection sufficiently to permit satisfactory sexual intercourse [1]. Erectile dysfunction is currently one of the most common sexual dysfunctions in men worldwide [2]. Despite the increasing demand for clinical services and the potential impact of ED and other sexual disorders on interpersonal relationships and quality of life, epidemiological data are relatively scarce [3]. Erectile dysfunction is usually underestimated in many developing countries, including Nigeria, because it is not a life threatening condition [2]. Also, due to the associated stigma, men with the problem rarely seek help [2]. Some believe that the condition would resolve on its own (primarily younger men) and others have the perception that ED is a normal part of aging (primarily older men). Approximately half of men aged 40 to 70 years are said to have some degree of ED [3]. The age-adjusted prevalence rates of ED among men attending primary care clinics was found to be 57.4% in Nigeria, 63.6% in Egypt, and 80.8% in Pakistan [4]. World-wide estimates of ED prevalence range from 2% in men younger than 40 years to 86% in men 80 years or older [5]. The most common medical conditions associated with erectile dysfunction are conditions that impair arterial blood flow to the erectile tissues or disrupt the neuronal circuitry. In a study of men in a hypertension center, ED was found to be highly prevalent and severe in men with hypertension [6]. Furthermore, ED was found to be a prognostic marker of the complications of hypertension namely: myocardial infarction and cerebrovascular accident [7]. Patients with diabetes mellitus have high rates of ED as a result of vascular disease and autonomic dysfunction. In the Massachusetts Male Aging Study (MMAS), the age-related probability of complete ED was three times greater in patients with diabetes than in those without diabetes [8]. Erectile dysfunction occurs at an earlier age in people with diabetes than in the general population [9]. Excessive and long-term use of a number of substances may also cause ED. Cigarette smoking has been shown to be associated with ED independent of smoking-related chronic illness [10, 11]. Recent studies have revealed that ED is not only a correlate of cardiovascular disease, diabetes, and metabolic syndrome; it is rather an early warning symptom [8].

Esposito et al. showed that lifestyle changes, such as weight loss and increased physical activity, were associated with improvement in sexual function in about one-third of men with ED [12]. Erectile dysfunction is an important public health problem that deserves increased support for basic science investigation and applied research. Embarrassment of patients and reluctance of both patients and healthcare providers to discuss sexual matters candidly contribute to under-diagnosis of ED [1]. For many men ED creates mental stress that affects their interactions with family and associates. Though many advances have occurred in both diagnosis and treatment of ED, its various aspects remain poorly understood by the general population and by most healthcare professionals. Ariba et al. [2] in a study to assess the perception and practices of primary care clinicians in the management of ED found that, though 76% of the respondents (primary care physicians) reported that ED was common in their practice, only 18% of them had ever prescribed any medication for the affected patients. Most of the clinicians (62%) would not take a sexual history unless the patient brought it up. This study attempts to add to the pool of information currently available from the developing world on the prevalence and risk factors of ED. We hope that the findings would increase awareness amongst men with ED and the primary care physicians who attend to majority of these affected men.

## Methods

The study was a descriptive cross-sectional population based survey among men aged 30-80 years in Ogbomoso, Nigeria from October 2014 to May 2015. Ogbomoso is located 100 km north of Ibadan, the Oyo State capital. The estimated population of the town is over 645,183 people spread over its five local government areas according to the Nigerian 2006 population census [13]. The indigenous people are of the Yoruba ethnic group. Majority of them are engaged in farming or trading. The study was approved by the Ethics Committee of LAUTECH Teaching Hospital, Ogbomoso. All participants gave written informed consent, and the procedures followed were in accordance with institutional guidelines. A multistage random sampling method was used. Ogbomoso North Local Government Area (LGA) was initially randomly selected out of the five LGAs that make up the city, five streets from the selected LGA were then randomly selected; from each of the streets consenting male adults aged 30-80 years living in different households were and interviewed. The questionnaires were administered to participants by well-trained research assistants. Participants’ demographic data (age, marital status, ethnicity etc.), cigarette smoking and alcohol intake were recorded. Other information regarding hypertension and medication administration, diabetes, heart disease and previous surgery (especially pelvic surgery) were asked for and documented. The instrument used was the International Index of Erectile Function-5 Questionnaire (IIEF-5) [3]. The International Index of Erectile Function-5 Questionnaire is a brief, reliable and valid self-administered questionnaire containing five domains, such as erectile function (questions 1 to 5,15), intercourse satisfaction (questions 6 to 8), orgasmic function (questions 9, 10), sexual desire (questions 11, 12), and overall satisfaction (questions 13, 14). It has been used widely in many countries, including Nigeria, to detect the presence and severity of ED [14]. The erectile function was classified based on the scores on IIEF-5 into severe 0-7; moderate 8-11; mild to moderate 12-16; mild ED;17-21, and 22-25, no dysfunction. Data analysis was done with the IBM- Statistical Package for Social Sciences (SPSS), version 20. Continuous variables were presented as means ±S.D. Categorical variables were expressed as frequencies and percentages. Unadjusted odds ratios of possible risk factors were calculated by univariate analyses. Binary logistic regression analysis was used to eliminate the effect of possible confounders on the risk factors to get the adjusted odds ratios. Variables with odds ratio (OR) > 1 and P value < 0.05 were considered significant risk factors. Confidence Interval (CI) of 95% was used.

## Results

Of the 243 men who participated in the study, two respondents were excluded from statistical analysis because of incomplete data. The results of the remaining 241 participants are here presented. Table 1 shows the socio-demographic characteristics of respondents. The mean age was 46.7 (±13.7) years while the age range was 30-80 years. Majority of the men were married (93.4%) while the others were either unmarried or separated. More than 90% of participants belonged to the Yoruba ethnic group while the rest belonged to the Igbo, Hausa and other Nigerian ethnic groups. More than half of respondents had at least secondary school education while the others had either primary or no formal education. Table 2 shows the prevalence and pattern of ED among respondents. The general prevalence of ED in this study was 58.9%. Sixty-seven (47.2%) respondents had mild ED, 16 (11.3%) respondents had moderate ED and 59(41.5%) respondents were found to have severe ED. Table 3 shows the unadjusted and adjusted odds ratios of the possible risk factors for ED. Age, hypertension, use of anti-hypertensive drugs, diabetes mellitus and heart disease all had significant unadjusted associations with ED: [OR = 2.42 (95% CI 1.36-4.29), P= 0.002)], [OR = 5.34 (95% CI 2.28-12.48), P = <0.0001], [OR = 5.5.46 (95% CI 2.21-13.52), P= <0.0001], [OR = 17.97(95% CI 2.38 - 135.67), P = <0.0001] and [OR= 5.25 (95% CI 1.51-18.17), P = 0.009] respectively. Following adjustment for potential confounders through binary logistic regression analysis, only diabetes mellitus, among the five possible risk factors included in the model, maintained a positive statistically significant relationship with ED [OR= 8.31(95%CI 1.02-67.65), P= 0.048]. Cigarette smoking, alcohol consumption, lack of regular exercise and previous history of surgery did not have statistically significant relationship with ED.

**Table 1:** Socio-demographic characteristics of respondents

Variables	Frequency (%)
**Age group**	
30 – 39	86 (35.7)
40 – 49	72 (29.9)
50 – 59	44 (18.3)
60 – 69	27 (11.2)
70 – 79	12 (5.0)
Mean Age = 46.7 ± 13.7	
**Marital Status**	
Married	225 (93.4)
Single	7 (2.9)
Divorced	3 (1.2)
Separated	6 (2.5)
**Ethnic Group**	
Yoruba	225 (93.4)
Ibo	10 (4.1)
Hausa	1 (0.4)
Others	5 (2.1)
**Educational Status**	
No Formal Education	29 (12.0)
Primary	48 (19.9)
Secondary	56 (23.2)
Post-Secondary	43 (17.8)
University	65 (27.0)

**Table 2:** Prevalence and pattern of erectile dysfunction among the respondents

Variable	Frequency (%)
**Prevalence of ED (n=241)**	
ED	142 (58.9)
No ED	99(40.7)
**Pattern of ED (n=142)**	
Mild	67(47.2)
Moderate	16(11.3)
Severe	59(41.5)

**Table 3:** Unadjusted and adjusted odds ratios of possible risk factors of erectile dysfunction among respondents

Risk Factors	No (%) positive	Unadjusted Odds Ratio	P value	Adjusted Odds ratio	P value
**Age**					
≤ 49 (n=158)	82(51.9)	1(reference)		1(reference)	
≥50 (n=83)	60(72.3)	**2.42 (1.36-4.29)**	**0.002**	1.56(0.84-2.88)	0.161
Smoking					
No (n=161)	89(55.3)	1(reference)			
Yes (n=80)	53(66.2)	1.59(0.93-1.88)	0.103		
**Alcohol**					
No (n=169)	108(63.9)	1(reference)			
Yes (n=72)	34(47.2)	0.51 (0.29-0.88)	0.016		
Exercise					
No (n=158)	96(60.8)	1.25 (0.73-2.13)	0.424		
Yes (n=83)	46(55.4)	1(reference)			
Hypertension					
No (n= 193)	101(52.3)	1(reference)			
Yes (n=48)	41(85.4)	**5.34 (2.28-12.48)**	**<0.0001**	2.35(0.24-23.58)	0.467
**Drugs for HTN**					
No (n=198)	105(53.0)	1(reference)			
Yes (n=43)	37(86.0)	**5.46 (2.21-13.52)**	**<0.0001**	1.36(0.12-15.49)	0.802
Diabetes mellitus					
No (n=218)	120(55.0)	1(reference)			
Yes (n=23)	22(95.7)	**17.97(2.38-135.67)**	**<0.0001**	**8.31(1.02-67.65)**	**0.048**
**Heart disease**					
No (n=218)	122(56.0)	1(reference)			
Yes (n=23)	20(87.0)	**5.25 (1.51-18.17)**	**0.009**	1.17(0.26-5.23)	0.833
Surgery					
No (n=223)	11(61.1)	1(reference)	0.844		
Yes (n=18)	131(58.7)				

## Discussion

In this study we got a prevalence of 58.9% for ED among men aged 30-80 years. This is similar to the findings in other studies in the same region and even outside Nigeria. In a study conducted at the University College Hospital (UCH), Ibadan (also in the south-western part of Nigeria), the prevalence of ED was 55.1% [14]. In the UCH study, the age range of the respondents was 18-70 years compared to 30-80 years in our study. This suggests that the respondents in the UCH study belonged to a younger age group and this might account for the little disparity in the findings. Also, our study was community based while the UCH study was hospital based. The 58.9% prevalence obtained in this study is also similar to the finding of the first community based study of ED (the MMAS) that got a prevalence of 52% [8]. The age range of the respondents in the MMAS was 40-70 years as against 30-80 years used in our study; this is a likely reason for the difference in results. However, the prevalence of ED found in a study conducted in Turkey was 69.2% [15]. It was also a population based study but men aged 40 years and above were interviewed. The significant disparity in the prevalence might be due to the fact that older men were assessed and the respondents self-rated themselves. We found in this study that increasing age had significant unadjusted association with ED, though; the adjusted relationship was not significant. Most of the studies cited, including the UCH, the MMAS and the Turkish studies, found a positive significant association between age and ED [14, 15]. The likelihood of ED increases with age but it is not an inevitable consequence of aging [1]. For the elderly and for others, ED may occur as a consequence of specific illnesses or of medical treatment for certain illnesses, resulting in fear, loss of image and self-confidence, and depression [1]. In men of all ages, erectile failure may diminish willingness to initiate sexual relationships because of fear of inadequate sexual performance or rejection [1]. Since males, especially older males, are particularly sensitive to the social support of intimate relationships, withdrawal from these relationships because of such fears may have a negative effect on their overall health [1]. We noted that about half of those with ED in this study had the mild type (47.2%). This observation is similar to the outcome of the Turkish study [15], and it might partly explain why most ED sufferers do not seek medical attention.

In this study, alcohol consumption was not found to be a significant risk of ED. Alcohol in small amounts improves erection and increases libido because of its vasodilatory effect and anxiety suppression; however, large amounts can result in central sedation, decreased libido, and transient erectile dysfunction. Chronic alcoholism can cause hypogonadism and polyneuropathy, which can affect penile nerve function [16]. In the Health Professionals Follow-up Study, moderate drinkers (1 or 2 drinks per day) had a lower prevalence of erectile dysfunction, compared with either heavy drinkers or nondrinkers [17]. In the Massachusetts Male Aging Study, a change in heavy drinking status was not associated with reduced risk of erectile dysfunction, suggesting that chronic heavy alcohol consumption might have an irreversible effect on erectile function because of neurological damage [18]. Why alcohol intake did not have a significant relationship with ED inour study cannot be immediately explained since we did not quantify the amount of alcohol taken by the participants. Hypertension had a significant unadjusted relationship with ED in this study, but the adjusted association was not significant. Erectile dysfunction is often found in hypertensive men, and several mechanisms could be implicated in its pathogenesis. Erectile dysfunction can be considered a symptom of damage to the vascular endothelium [9]. Nitric oxide synthesis is necessary to stimulate smooth muscle relaxation and to increase blood flow to levels appropriate for an erection; however, it is inhibited through age, disease, and behavior-related pathways. Severe hypertension is related to endothelial dysfunction with reduced nitric oxide production by the endothelium [19]. Thus, severity of hypertension might negatively affect erectile function through reduced nitric oxide production. Diabetes was found to be a significant risk factor for developing ED in this study, even after adjustment for potential confounders. In the MMAS sample, the age related probability of complete ED was three times greater in patients with diabetes than in those without diabetes [8]. Other studies using diabetic populations have consistently found a high prevalence of diabetes-related ED, with estimates ranging from 35 to 50% and up to 75% [8]. The prevalence of ED in patients with diabetes has been reported to increase from 15% in men aged 30 to 35 years to 55% in men aged 60 years [8]. Erectile dysfunction occurs at an earlier age in people with diabetes than in the general population and often follows, or leads to, the diagnosis of either type 1 or type 2 diabetes [20]. We found no statistically significant association between cigarette smoking and ED. This is in tandem with the MMAS that did not show a significant difference in cases of ED between current smokers and nonsmokers [8]. However, the association of ED with certain risk factors was greatly amplified in current smokers in the MMAS. According to MMAS data analysis, the age-adjusted probability of complete ED in participants treated for heart disease was 56% for current smokers compared to 21% for nonsmokers [8]. A review of 18 studies by Dorey concluded that tobacco smoking has adverse effects on erectile function, with smokers being approximately 1.5 times more likely to report ED compared with nonsmokers [21]. A cross-sectional study conducted in Italy among men who were 18 years and above to compare the risk of ED in nonsmokers with current smokers and ex-smokers in 2010 yielded odds ratios of 1.7 and 1.6 respectively [11]. The study also showed that the risk of developing ED is influenced by smoking and that the duration of the habit increases this risk [11]. Variation in the quantity of tobacco smoked per day and the duration of smoking among the populations where our study and these cited studies were conducted may account for the observed disparities. Though increasing age, hypertension, use of antihypertensive drugs and heart disease did not have significant adjusted association with ED, the fact that they all had unadjusted positive association with the ailment could mean that the development of ED in the study population has a multifactorial etiology.

## Conclusion

We conclude that the prevalence of ED is high among Nigerian male adults of south-western origin and diabetes mellitus is a very important risk factor in them. It is a known fact that ED sufferers often do not seek for medical help but usually suffer in silence because of the stigma associated with the condition. It is our belief that sufferers of ED need not suffer in silence because effective medical help is available to them. Physicians, especially primary care ones, need to pay more attention to the sexual history of their patients in order to diagnose and manage ED more frequently. There is need for public education/enlightenment by government and advocacy groups to address the problems of ignorance and stigmatization associated with erectile dysfunction. This will go a long way to ameliorate the psychosocial burden of the disease on the sufferers.

### What is known about this topic

- Increasing age, hypertension, side effects of drugs, diabetes mellitus, heart disease, pelvic surgery etc. are all know risk factors for ED;
- Erectile dysfunction occurs at an earlier age in people with diabetes than in the general population;
- There is an associated stigma with the condition and this explains why men with the condition rarely seek help.

### What this study adds

- Diabetes mellitus seems to be the most important risk factor among our study population
- Cigarette smoking and alcohol consumption seem not to be important risk factors among our study population as compared to a number of previous studies
- It is a population based study from Africa.
